# Learning in a noisy world: How lucky successes and unlucky failures shape learning consequences

**DOI:** 10.1371/journal.pone.0352416

**Published:** 2026-06-29

**Authors:** Amanda Zaidan Chen, Martha Jeong, Michele Rigolizzo

**Affiliations:** 1 Department of Management, The Hong Kong University of Science & Technology, Hong Kong; 2 Department of Management, Montclair State University, Montclair, New Jersey, United States of America; University of Trento: Universita degli Studi di Trento, ITALY

## Abstract

Learning is noisy. People can face *unlucky failure*, in which they fail despite using effective strategies, or experience *lucky success*, in which they succeed in spite of using suboptimal strategies. Both situations can serve as potentially valuable learning opportunities, as long as performance outcomes are interpreted accurately. This report examines people’s ability to recognize and adjust for these errors during the learning process. Our pilot data demonstrated that people assumed that noise affected failure at greater rates, and thereby believed successful outcomes were more informative learning cues. Based on these findings, our research aims to investigate whether experiencing a lucky success is more detrimental to future learning than experiencing unlucky failure, especially when it occurs at the beginning of a learning process. In other words, we seek to understand whether learners sufficiently discount the role of noise in successful outcomes or place too much weight on the informative value of their success, thereby hindering their learning when these outcomes are noisy.

## Introduction

The world is noisy – people can fail despite doing the right thing or stumble backwards into success. Learning requires that people cut through this noise, to accurately interpret their performance outcomes and determine which strategies are truly effective. If people realize with additional feedback that their failure was unlucky (i.e., failure was due to randomness), they should attempt the same strategy again, instead of unnecessarily testing alternative strategies. On the other hand, if people recognize with subsequent learning opportunities that their success was lucky (i.e., success was due to randomness), they should search for more effective strategies. In line with experiential and reinforcement learning theories [1–5,6] and research on after-event reviews [7], we rely on the definition of learning as a process of accurate belief updating that leads to behavioral change, specifically, revising one’s beliefs about effective strategies based on performance feedback and adjusting behavior accordingly. We argue, however, that people’s ability to recognize noise and learn accurately is not uniform, but depends critically on whether the noisy outcome takes the form of a lucky success or an unlucky failure.

As people cannot always distinguish effective behaviors from ineffective ones ex ante, they often work backwards based on performance outcomes [7–9]. Reinforcement learning theory would suggest that people learn by repeating behaviors that led to success and revising behaviors that led to failure, updating their beliefs in proportion to the discrepancy between expected and actual outcomes [5]. Consistent with this approach, research on after-event reviews describes how people investigate past experiences to determine what behaviors led to subsequent outcomes, as a way to improve future performance [7,10–14]. Both frameworks assume true and consistent causal relationships exist between behaviors and outcomes [7,14]. This assumption, however, starts to weaken in a noisy world, in which lucky success and unlucky failure can obscure the causal link between behavior and outcome, resulting in feedback that can be potentially misleading.

Our research investigates variation in people’s ability to learn from two types of noisy performance outcomes: lucky success versus unlucky failure. This can occur for situations involving multiple rounds of learning wherein, at some point, the outcomes can be inaccurate. If people can identify the inaccurate feedback as an outlier, their learning will remain unaffected. However, research shows that people engage with success and failure in fundamentally different ways [15,16]. The affective responses to these performance outcomes can impact learning since success feels ego-affirming, whereas failure is more often ego-threatening [17]. Therefore, we expect lucky success and unlucky failure to affect learning in systematically different ways.

The asymmetry in learners’ reactions is further deepened by the fundamental attribution error. Specifically, because people tend to attribute success internally and failure externally [18,19], we expect people to be more likely to view successful outcomes as true reflections of their own behavior and failed outcomes as the result of external factors beyond their control. As a result, people can assume that successful outcomes are more reliable indicators of optimal behavior than failed outcomes are of suboptimal behavior, a tendency that is consistent with research showing that people generally perceive success as being more useful than failure [20–24]. We define the useful nature of a performance outcome as the degree to which it serves as a reliable signal of strategy effectiveness. Altogether we argue that people treat success as more diagnostically valuable than failure, making lucky success particularly difficult to identify (and dismiss) as a noisy outlier, while unlucky failure is more readily discounted as unrepresentative of true performance.

After people receive feedback that their behavior resulted in a successful outcome, we predict they will anchor on the belief that the behavior is actually effective, while overlooking the possibility of noise. Indeed, research has shown people experiencing success feel less motivated to learn and adapt their behaviors [9]; stop learning as they feel confident and complacent following old routines [25–28]; and tend to repeat behaviors leading to success [12]. Despite additional feedback suggesting their prior success was due to randomness, we believe that lucky success will be weighted more heavily than it should be, instead of being properly discounted in the learning process. Moreover, we expect this effect to be particularly pronounced when the lucky success occurs at the beginning of a learning sequence. Early outcomes generate larger belief updates as outcome expectations are still uncertain [29,30], meaning a lucky success at the start of a learning sequence could trigger a stronger and more erroneous shift in beliefs than one occurring later. Additionally, early in a learning sequence, people have less experience to draw on and a reduced ability to differentiate noise from accurate feedback, making them even less likely to recognize the lucky success as an outlier and more likely to anchor on it as a true signal of effective behavior.

In contrast, when faced with failure, we predict people will be more likely to acknowledge the possibility of noise and therefore be less likely to automatically believe they did the wrong thing. In situations in which failure occurs despite using a strategy believed to be effective, we expect that individuals will be more willing to disregard this noisy signal and continue learning from additional feedback opportunities. In this way, we do not expect differences based on the timing of the unlucky failure because we predict people are more likely to disregard the failure as an outlier whether it comes at the beginning or end of a learning sequence.

H1: The occurrence of lucky success will be more detrimental to learning as compared to unlucky failure.

H2: The learning disadvantages associated with lucky success will be strongest when it occurs earlier in a learning sequence rather than later.

## Methods

### Ethics Statement

All authors declare no conflicts of interest. No artificial intelligence was used in this research or the creation of this article. This research received approval from the governing institution’s ethics board (HREP-2023–0312). All participants provided informed consent electronically before beginning the study, and were informed of their right to withdraw at any time.

### Pilot Studies

With two pilot studies, we surveyed lay beliefs regarding how likely performance outcomes were affected by noise and how informative people believed them to be. Please refer to S1 Appendix for all stimuli and measures used in the pilot studies (Section A for Pilot A, Section B for Pilot B).

### Pilot A

*Participants*. We recruited Prolific workers (*N* = 200, *M*_*age*_ = 45.13 years, *SD* = 13.96 years; 45.0% male) to participate in an academic study about predictions on November 19, 2025.

*Design and procedure*. We described two scenarios to all participants (with the order counterbalanced) – in which an individual succeeded despite using suboptimal strategies (labeled as “lucky success”) and an individual failed despite using optimal strategies (labeled as “unlucky failure”). We asked participants to estimate what % of the time these situations occurred on a 0–100 scale from “*This never happens at all*” to “*This happens all the time*.”

## Results

A paired-samples t-test revealed no significant mean difference in the occurrence of lucky success versus unlucky failure (32% versus 33%, respectively, *t*(199) = .798, *p* = .426). Average predictions, however, are not able to shed light on the distribution of people’s beliefs. We were specifically interested in the presence of skewed beliefs, specifically whether people believed that unlucky failure was more common than lucky success (or vice versa). We therefore categorized participants’ predictions by their within-person difference in beliefs between performance outcomes, and an interesting pattern emerged.

**Fig 1:**
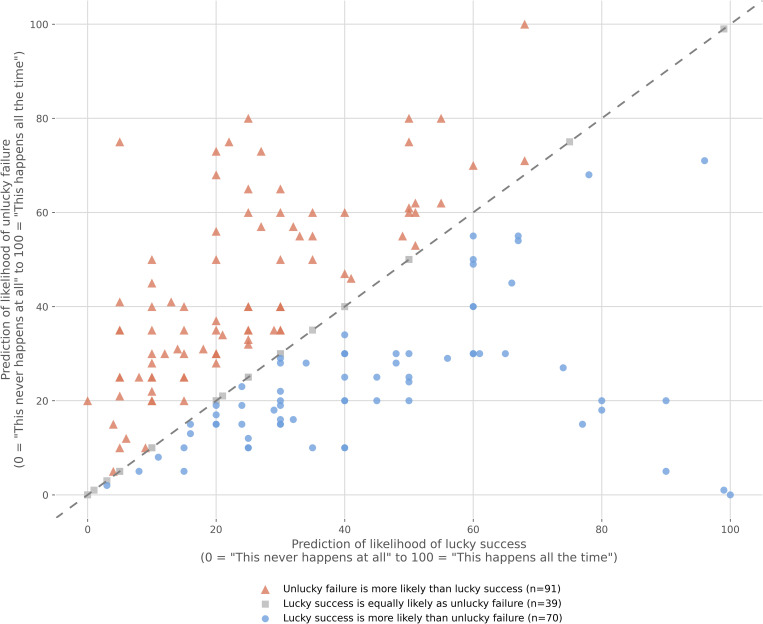
Distribution of individual beliefs in noise predictions.

If a participant believed that unlucky failures were more likely to occur than lucky successes, we categorized this as 0 (representing beliefs that noise was more common with failures). If a participant believed both occurred at similar rates, we categorized this as 1 (representing beliefs that noise was equally likely to affect both performance outcomes). If a participant believed that lucky successes were more likely to occur than unlucky failures, we categorized this as 2 (representing beliefs that noise was more common with successes). Looking at this within-person categorization, we found that 45% believed that unlucky failure was more likely than lucky success while 36% believed the opposite to be true (i.e., lucky success was more likely than unlucky failure), with 19% predicting both to occur at similar rates, *x*^2^(2, 197) = 20.23, *p* < .001. In this way, we found initial evidence suggesting most people hold uneven predictions of how likely their performance outcomes are affected by noise, with a greater number believing it is more common for their failures to occur as a result of bad luck, than their successes to occur as a result of good luck (Fig 1).

### Pilot B

*Participants*. We recruited Amazon’s Mechanical Turk workers (*N* = 412, *M*_*age*_ = 43.57 years, *SD* = 12.75 years; 44.5% male) to participate in an academic study on negotiations on August 8, 2024.

*Design and procedure*. We told all participants their task was to read a negotiation message (requesting a 20% discount from the seller) and consider why the message had succeeded or failed in receiving a discount. In reality, all participants read the same negotiation message and were randomly assigned to learn the message had either succeeded or failed in receiving the desired discount. We asked participants two questions: 1) how informative they believed the message was in learning how to communicate effectively in this negotiation setting; and 2) how certain they felt they learned the pattern that made the message succeed/fail in receiving the discount. Both questions were asked on a Likert scale from 1 (Not at all) to 5 (Extremely).

## Results

Participants believed messages that succeeded in receiving the discount to be more informative in learning how to effectively communicate in this negotiation than messages that failed in receiving the discount (*M*_*succ*_ = 3.46, SD = .900 vs. *M*_*fail*_ = 2.71, SD = .916, t(402) = 8.23, *p* < .001). Additionally, participants felt more certain they had identified why a message had succeeded versus failed (*M*_*succ*_ = 3.21, SD = .993 vs. *M*_*fail*_ = 2.61, SD = 1.06, t(401) = 5.86, *p* < .001). In other words, those who were told the message had succeeded felt more certain they learned why the message was successful in contrast to those who were told the message had failed felt certain they learned why the message was unsuccessful.

We acknowledge the possibility the informativeness measure, designed to capture beliefs about the diagnostic value of the message, could have been interpreted in alternative ways, such as deviation from what was expected or how much uncertainty it reduced. Importantly, our evidential interpretation was more directly supported by the second measure, namely participants’ certainty that they had identified why the message succeeded or failed, which captured attribution of causal effectiveness rather than mere uncertainty reduction. Moreover, in our proposed studies, we plan to measure learning directly by having participants perform tasks, receive outcome feedback, and then directly identify effective strategies.

## Discussion

Our two pilot results presented an interesting phenomenon. As a greater number of people believed that noise was more likely to affect failure compared to success (i.e., experiencing a lucky success was more rare), then we should accordingly expect people to believe successes are more useful because they are more likely to be true signals of effective behavior. Indeed, we found this to be the case. When confronted with identical stimuli, participants reacted differently depending on the performance outcome. Participants believed stimuli they were told led to success to be more informative in learning how to communicate effectively, and felt more certain of their learning than identical stimuli they were told led to failure. These results provided initial evidence to suggest that noise generated by lucky success can be more detrimental to learning than noise caused by unlucky failure, as people assume the occurrence of lucky success is rare and thereby consider successful outcomes as stronger diagnostic signals of performance, more likely overlooking the possibility of noise.

### Proposed Studies

#### Study 1.

Building on our initial pilot study results, we now propose a formal test comparing learning from experiencing a lucky success versus an unlucky failure, in which learning represents the process of accurate belief updating. We plan to document differences in learning through both quantitative and qualitative measures. We will give all participants the opportunity to learn from three learning trials, in which the last two trials are true performance outcomes (i.e., effective behaviors lead to success and ineffective behaviors lead to failure). While the existence of a noisy signal (i.e., effective behaviors lead to failure and ineffective behaviors lead to success) should impair learning more than not having one, we expect that people are more likely to recover from the occurrence of an unlucky failure than a lucky success. In other words, we predict less learning when people experience lucky success versus unlucky failure. Please refer to S1 Appendix, Section C for all stimuli and measures.

*Participants.* We plan to recruit 800 U.S. resident participants on the Prolific platform to participate in an academic study on negotiation. We plan to recruit participants with approval ratings over 98% (with a minimum of 500 previous submissions) who indicate English fluency; list English as their primary language; pass a basic attention check; and have not participated in similar studies. Our proposed sample size is based on recruiting 150–200 participants per condition for between-subjects behavioral research [31], resulting in a target sample of 800 participants (200 per condition). A power analysis indicates this sample size provides over 90% power to detect a small-to-medium effect (w = .15, α = .05) on our primary Chi-square test and should retain adequate power (> 80%) even after anticipated exclusions due to failed attention checks or prior study participation.

*Design and procedure*. We will instruct all participants their task is to engage in a negotiation simulation, in which they play the role of the buyer. The goal is to identify the communication style that is most effective for receiving a discount from the seller. We will ask participants to do three practice trials in which they receive feedback before they do their actual negotiation simulation. We plan to define learning in this study as recognition that use of a “tough” communication style is more effective at securing discounts than use of a “warm” communication style in the context of online distributive negotiations, borrowing findings and stimuli from prior research [32].

We plan to randomly assign participants to one of four conditions, in which each condition represents a different learning sequence (Table 1):

**Table 1:** Design of the four experimental learning sequence conditions (Study 1).

	Trial 1	Trial 2	Trial 3
**Condition 1**	True Success	True Success	True Success
**Condition 2**	***Unlucky Failure***	True Success	True Success
**Condition 3**	True Failure	True Failure	True Failure
**Condition 4**	***Lucky Success***	True Failure	True Failure

1. Condition 1 – three true successes (i.e., effective tough strategy is used three times and succeeds each time);
2. Condition 2 – unlucky failure followed by two true successes (i.e., effective tough strategy fails once followed by succeeding twice);
3. Condition 3 – three true failures (i.e., ineffective warm strategy is used three times and fails each time); or
4. Condition 4 – lucky success followed by two true failures (i.e., ineffective warm strategy succeeds once followed by failing twice).

For ease of reference, we will refer to the conditions by the first learning trial participants experience and therefore label the conditions as: true success (Condition 1), unlucky failure (Condition 2), true failure (Condition 3), and lucky success (Condition 4). In the first two conditions, participants experience the effects of using a tough communication strategy and in the last two conditions, participants experience the effects of using a warm communication strategy. With this design, we can test whether the occurrence of a lucky success or unlucky failure at the beginning of a learning sequence differentially affects downstream learning in two different ways. First, we can compare difference in learning between Conditions 2 and 4 (comparing unlucky failure versus lucky success). While we acknowledge the potential limitation of this analysis as participants in these two conditions are learning from different stimuli (i.e., different communication strategies), this represents one way to test a direct comparison between the two noisy signals. Second, we can compare the gap in learning performance between Conditions 1 & 2 versus Conditions 3 & 4 to help address the limitation described above. We would therefore predict a greater difference between Conditions 3 & 4 versus Conditions 1 & 2, as we believe lucky success to be more detrimental for learning than unlucky failure.

As an exploratory measure, we plan to measure the time participants spend reviewing their learning stimuli. We also plan to ask participants how useful the trials were for their learning on a Likert scale from 1 (Not useful at all) to 5 (A great deal useful).

After the practice trials, we will ask three questions to test for learning, which is recognizing the effective use of a “tough and firm” communication style in this negotiation simulation.

There are two quantitative measures of learning. First, we will display two negotiation messages with the same offer price in which one message utilizes a warm communication strategy while the other utilizes a tough communication strategy and ask participants to select the message they believe will give them the best chance of the seller agreeing to their requested discount. Second, we will directly ask participants what kind of communication strategy they believe is more effective in this negotiation context on a Likert scale of 1 (Very warm and friendly) to 5 (Very tough and firm).

Next, as a qualitative measure of learning, we will instruct participants to engage in the actual simulation and write their own negotiation message to the seller, given what they have learned from their practice trials. We will show all participants an advertisement for a phone listed for $500 and instruct participants to write a message to the seller offering $400. We will incentivize participants by offering a bonus opportunity for those who write effective negotiation messages that are able to secure full discounts from the seller. As an exploratory measure, we plan to ask how confident they are their message will receive the full discount from the seller on a Likert scale from 1 (Not at all) to 5 (Extremely confident).

### Proposed Analyses

Our main DV is difference in learning as a function of what learning trials participants are exposed to, in which we are specifically interested in the experience of a lucky success versus an unlucky failure. We measure and analyze learning in multiple ways.

We begin with the proposed analyses for the two quantitative measures of learning. First, for the question in which participants are asked to select between two negotiation messages, we will test for differences in selection of the effective message (the one utilizing a tough communication style) using a Chi-square test, generally as function of condition and then specifically comparing our two conditions of interest (Condition 2 versus 4). We then plan to create two variables – Stimuli (Ineffective Warm Messages/Effective Tough Messages) and Noise (No/Yes) in order to run a logistic regression predicting selection of the effective message from Stimuli, Noise, and their interaction. We predict a positive interaction, such that noise in the form of lucky success is more detrimental to learning than noise in the form of unlucky failure.

Second, for the other quantitative measure in which the effectiveness of communication style type is directly asked (as a continuous variable), we will test for learning differences in two ways. In order to directly compare the difference between Condition 2 (unlucky failure) versus Condition 4 (lucky success), we will run a one-way ANOVA with post-hoc analysis directly comparing these two conditions. We predict that learning will be more negatively affected by exposure to lucky success than unlucky failure, so we make the specific prediction that Condition 4 participants (lucky success) will be less likely to correctly recognize the tough communication style as being effective in this simulation than Condition 2 participants (unlucky failure). Next, we will estimate an OLS regression with Stimuli, Noise, and their interaction as predictors. Again, we expect a positive interaction. In other words, we predict a smaller performance gap in participants’ ability to recognize the correct communication style between Conditions 1 & 2 (true success vs. unlucky failure) and predict a larger gap when it comes to Conditions 3 & 4 (true failure vs. lucky success).

For our qualitative measure of learning, we plan to analyze participants’ use of a “tough and firm” communication style in their written negotiation messages using the same natural language processing algorithm developed by the original authors which documented the advantages of using a “tough” communication style in an online distributive negotiation [32]. Specifically, we plan to test for politeness differences in participants’ negotiation messages as a function of condition using a well-established, open-source text analysis R package [33]. We plan to conduct the analysis in the same two ways described above with the continuous quantitative measure of learning.

As exploratory measures, we plan to test for any differences in time spent reviewing the learning stimuli, perceptions of the usefulness of the learning trials, and perceptions of confidence as a function of condition with one-way ANOVAs (with post-hoc analyses).

#### Study 2.

We also plan to test whether the timing of the noisy signal matters. Is lucky success only harmful when it occurs at the beginning of a learning sequence or is its mere presence harmful regardless of when it occurs? We predict that lucky success is particularly detrimental to learning when it occurs at the beginning. On the other hand, we expect that unlucky failure is in general less harmful, and we furthermore believe there will be little difference in learning regardless of when it occurs. If this is true, our results would suggest that it’s not merely an anchoring effect, in which early noisy signals in the learning sequence are harmful, but something specific about lucky success that is particularly troublesome, especially when it occurs early in a learning sequence. Please refer to S1 Appendix, Section C for all stimuli and measures.

*Participants.* We plan to recruit 800 U.S. resident participants on the Prolific platform to participate in an academic study on negotiation. We plan to recruit participants with approval ratings over 98% (with a minimum of 500 previous submissions) who indicate English fluency; list English as their primary language; pass a basic attention check; and have not participated in similar studies. The sample size calculation follows the same convention as proposed Study 1, given the same 4-condition design.

*Design and procedure*. We plan to utilize the same design as proposed Study 1, to keep the learning stimuli consistent and learning results comparable. The only difference is that, given our interest in studying order effects, we plan to have all participants exposed to a noisy signal that either comes at the end or beginning.

Specifically, participants will be randomly assigned to one of four conditions that vary by type of noise (unlucky failure vs. lucky success) and timing (at the beginning vs. at the end) (Table 2). These manipulations will be presented as different learning sequences, which will be characterized by:

**Table 2:** Design of the four experimental learning sequence conditions (Study 2).

	Trial 1	Trial 2	Trial 3
**Condition 1**	True Success	True Success	***Unlucky Failure***
**Condition 2**	***Unlucky Failure***	True Success	True Success
**Condition 3**	True Failure	True Failure	***Lucky Success***
**Condition 4**	***Lucky Success***	True Failure	True Failure

1. Condition 1 – two true successes followed by an unlucky failure (i.e., effective tough strategy succeeds twice then fails);
2. Condition 2 – unlucky failure followed by two true successes (i.e., same as Condition 1 but the noise occurs at the beginning);
3. Condition 3 – two true failures followed by a lucky success (i.e., ineffective warm strategy fails twice then succeeds); or
4. Condition 4 – lucky success followed by two true failures (i.e., same as Condition 3 but the noise occurs at the beginning).

For ease of reference, we plan to refer to the four conditions by learning violation type and order, so there is unlucky failure last (Condition 1); unlucky failure first (Condition 2); lucky success last (Condition 3); and lucky success first (Condition 4).

In this way, two of the conditions (Conditions 2 and 4) will be exact replications of what we test in Study 1 (as the two examples of noisy signals in Conditions 2 and 4), and the other conditions (Conditions 1 and 3) contain the same information, respectively, but with the noisy signal occurring at the end. Another way to think about the conditions is that for Conditions 1 and 2, participants are shown three messages that use the effective tough strategy that succeeds twice then (unluckily) fails once (either at the end or beginning). And for Conditions 3 and 4, participants are shown three messages that use the ineffective warm strategy that fails twice then (luckily) succeeds once (either at the end or beginning). We plan to ask the same two quantitative measures of learning and exploratory measures as Study 1.

### Proposed Analyses

To analyze the two quantitative measures of learning, we plan to create two variables – Type of Noise (Unlucky Failure/Lucky Success) and Timing (End/Beginning). For the first quantitative measure of learning in which participants are asked to select between two negotiation messages, we will first test for the main effect of the type of noise using a Chi-square analysis. We will then run a logistic regression predicting selection of the effective message from Type of Noise, Timing, and their interaction. We predict that lucky success will be more detrimental for learning than unlucky failure, in which the timing of the noise affects the impact of lucky success to a greater extent than it affects the impact of unlucky failure.

For the second quantitative measure in which effective communication strategy is directly asked, we will first examine the main effect of type of noise with a one-tailed t-test. Then we will estimate an OLS regression with Type of Noise, Timing, and their interaction as predictors. We predict a negative interaction term, which suggests a smaller performance gap in participants’ ability to recognize the effective communication style between Conditions 1 & 2 (unlucky failure at end or beginning) and a larger gap when it comes to Conditions 3 & 4 (lucky success at end or beginning).

As exploratory measures, we plan to test for any differences in time spent reviewing the learning stimuli, perceptions of the usefulness of the learning trials, and perceptions of confidence using an OLS regression with Type of Noise, Timing, and their interaction as predictors.

## Discussion

The proposed research examines a fundamental but underexplored aspect of human learning, namely the ability to recognize and adjust for noise in performance outcomes. By investigating how lucky success and unlucky failure differentially interfere with the learning process, we aim to shed light on why people sometimes do not learn effectively from experience even when feedback is available. Our expected results would suggest that the problem is not simply that learning is noisy, but that people are systematically more vulnerable to certain types of noise than others. Understanding this asymmetry has broad implications for a variety of learning contexts in which people rely on performance feedback to guide their future behavior, and points to the importance of considering not just whether performance feedback is available, but how people are likely to interpret and act on it.

Our paper contributes to research in three ways. First, we contribute to literature on experiential learning by examining the effect of randomness in the process of learning. Experiential learning is powerful, but less controlled than information-based or classroom learning. As individuals try approaches and observe outcomes, they must infer, rather than being explicitly told, what strategies are effective. In this way, experiential learning in real-world settings introduces noise into the learning process. Our work investigates whether and how that noise impacts learning trajectories. Second, we integrate work on decision-making with learning by showing the impact of the fundamental attribution error on experiential learning. Even though failure is often more information rich than success, people are more likely to dismiss the failure as unlucky and assume the success as indicative of their learning about effective strategies. Third, we are the first paper we know of to examine the role of the timing of inaccurate feedback in experiential learning. If it is simply noise that is problematic because people update their beliefs even in the face of inaccurate feedback, then the timing of those outcomes should not matter. However, if our results demonstrate that early lucky success is particularly detrimental to learning, this means that learners anchor on success and are more subject to confirmation bias after success than failure. In other words, they are more likely to interpret subsequent outcomes in light of the early success. This is mitigated if the lucky success occurs later because learners have already processed more information that contradicts the outcome.

From a practical standpoint, our expected results suggest that people can improve learning by responding more skeptically to successful outcomes. If the tendency is to believe them outright, organizations can introduce procedures for pausing and testing its diagnostic value against additional feedback rather than automatically (and potentially erroneously) assuming effective strategies have been implemented. In conclusion, our paper would thus propose that learners would benefit by exercising caution when it comes to early success and consider re-framing “beginner’s luck” to potentially be a “novice’s error.”

### Open Science Statement

All data, experimental materials, and analysis codes will be made publicly available upon study completion via ResearchBox, in accordance with PLOS ONE’s data availability policy. All proposed studies will be preregistered on AsPredicted prior to the onset of data collection.

## Supporting information

S1 Appendix Supplementary stimuli and measures. Contains the full stimuli and measures used in Pilot A (Section A), Pilot B (Section B), and the proposed Studies 1 and 2 (Section C).
(DOCX)
