# Supplementary material for: Learning in a noisy world: How lucky successes and unlucky failures shape learning consequences
Source: PLoS One. 2026 Jun 29;21(6):e0352416. doi: 10.1371/journal.pone.0352416 (PMC13313345; doi:10.1371/journal.pone.0352416)
Supplement: S1 Appendix — Contains the full stimuli and measures used in Pilot A (Section A), Pilot B (Section B), and the proposed Studies 1 and 2 (Section C). (DOCX) [file pone.0352416.s001.docx]

**S1** **Appendix. Supplementary stimuli and measures**

**Section A: Pilot A Stimuli & Measures**

**Section B: Pilot B Stimuli & Measures**

**Section C: Proposed Studies 1 & 2 Stimuli & Measures**

**Section A: Pilot A Stimuli & Measures**

**Stimuli & Measures:**

Participants were presented with the instructional prompts for “Lucky Success” & Unlucky Failure in a counter-balanced order.

Lucky Success: **“In life, sometimes you succeed simply due to good luck. These successes occur even when the strategy you use is suboptimal. So if you were to try the same strategy again, it would probably fail. We can label these events as lucky successes. These lucky successes are due to uncontrollable external factors and randomness.**

Here are some examples:

- Someone can make relatively weak arguments, and yet win a debate.
- Someone can design a product after incomplete market research, and have the product launch succeed.

**Among all successes, what percentage of the time do you think lucky successes occur?**0 represents 0% likelihood (i.e. "This never happens at all") and 100 represents 100% likelihood? (i.e. "This happens all the time").” Participants were then presented with a 0-100 sliding scale and were asked to write a few sentences supporting their estimate in a textbox.

Unlucky Failure: “**In life, sometimes you fail simply due to bad luck. These failures can occur even when the strategy you use is optimal. So if you were to try the same strategy again, it would probably work. We can label these events as unlucky failures. These unlucky failures are due to uncontrollable external factors and randomness.**

Here are some examples:

- Someone can make relatively strong arguments, but lose a debate.
- Someone can design a product after thorough market research, but have the product launch fail.

**Among all failures, what percentage of the time do you think unlucky failures occur?**0 represents 0% likelihood (i.e. "This never happens at all") and 100 represents 100% likelihood? (i.e. "This happens all the time").” Participants were then presented with a 0-100 sliding scale and were asked to write a few sentences supporting their estimate in a textbox.

**Section B: Pilot B Stimuli & Measures**

**Stimuli:**

“In this study, we will you show a negotiation message that a buyer sent to a seller.

The seller has posted a Craigslist ad for a used iPhone. The buyer has sent a message to the seller asking for a 20% discount.

These are real negotiation messages that buyers have sent and we will let you know whether the buyer succeeded or failed in receiving the discount.

Your task is to read the negotiation message and think about WHY the message succeeded or failed in getting the discount.”

Participants read one of the three messages below, but were randomly assigned to believe the message they read succeeded/failed in receiving the 20% discount from the seller.

- “Hi there - I’m happy to see your post about the phone. This iPhone matches what I wanted to buy. I’m willing to pay $125. Given the prices on similar phones currently for sale, I’m firm on that price. Let me know by tomorrow if the price is ok for you or else I’ll move on. I live in the area and I can come to meet you anywhere that is convenient for you. Thank you so much for your time and consideration. Hope you have a wonderful day. Sincerely, XXX”
- “Hello! I liked your listing and I am interested in buying the used iPhone. However, the asking price is too high for me. Instead, I am offering to pay $125. Does that work? If so, I look forward to doing business with you. If you want to sell your phone, let me know by tomorrow and I can pay in cash when I pick it up. I can meet you at a time and place that is convenient for you. I look forward to your consideration of my offer. Thanks again!”
- “Hello, I was looking at your post and this phone could meet my needs. I would be interested in taking this off your hands. I am willing to pick it up from you, but your asking price is too high for what you are offering. I am willing to pay $125 in cash for the phone. That’s my absolute limit, non-negotiable. And I can meet you whenever is most convenient for your schedule. Let me know if this will work for you and have a great day.”

Participants were asked to consider why the message succeeded/failed in getting the discount and selected reasons from a drop down list.

Reasons:

- The buyer’s communication style was warm and friendly.
- The buyer appeared to be an experienced/inexperienced negotiator.
- The buyer made the delivery convenient/inconvenient.
- The buyer appeared/didn’t appear sincere in making the transaction happen.
- The buyer’s communication style was tough and firm.

**Measures:**

*Informativeness*. “Reviewing this message that succeeded in/failed to receive a discount was informative in learning how to effectively communicate in this negotiation setting” [Answered on a Likert scale from 1 (Not at all informative) to 5 (Extremely informative].

*Certainty*. “I feel certain that I learned the pattern that made this negotiation message successful/fail in receiving the discount” [Answered on a Likert scale from 1 (Not at all certain) to 5 (Extremely certain)].

**Section C: Proposed Studies 1 & 2 Stimuli & Measures**

**Stimuli:**

“Imagine that you are negotiating to purchase a phone online. You found the phone you want, but you want to receive a discount from the seller. You have decided to send a message to the seller asking for a 20% discount. The phone is listed for $500, so you will offer $400. You are trying to decide how to communicate the offer so that you can get the discount you want. We will show you three different messages that were actually sent from buyers to real sellers on Craigslist. We will also tell you whether the buyer was able to receive the discount or not. Please read the messages carefully to help you think about how to write your own message. Research has shown that communication style can substantially affect negotiation outcomes. We can often learn a lot about what communication style is effective by studying messages that were sent asking for the same discount on the same product in the same marketplace.”

In Proposed Study 1, participants will be randomly assigned to one of four conditions.

| Condition | Trial 1 | Trial 2 | Trial 3 |
| --- | --- | --- | --- |
| 1 | True Success | True Success | True Success |
| 2 | ***Unlucky Failure*** | True Success | True Success |
| 3 | True Failure | True Failure | True Failure |
| 4 | ***Lucky Success*** | True Failure | True Failure |

In Proposed Study 2, participants will be randomly assigned to one of four conditions. Conditions 2 & 4 are identical between Proposed Studies 1 & 2.

| Condition | Trial 1 | Trial 2 | Trial 3 |
| --- | --- | --- | --- |
| 1 | True Success | True Success | ***Unlucky Failure*** |
| 2 | ***Unlucky Failure*** | True Success | True Success |
| 3 | True Failure | True Failure | ***Lucky Success*** |
| 4 | ***Lucky Success*** | True Failure | True Failure |

The messages for True Success (where the effective tough communication strategy succeeds in receiving the discount):

- “I want the phone but will only pay $115 for it. I will only pay that because its a used phone.  I think this is a reasonable price.”
- “Hello. I am interested in buying your phone. I don't like to haggle so I will give you my best offer of $115 dollars. It's the best I can offer and my only offer. Let me know if you are interested in selling for that price.”
- “I viewed your phone online and it seems satisfactory. I have alloted a specific amount I will spend on the phone. I am writing to let you know I can purchase the phone immediately with cash. I am prepared to give you $115 in cash, but I must meet you today or I will pursue two other options.”

The messages for True Failure (where the ineffective warm strategy fails in receiving the discount):

- “Hello there! I really admire your post about the IPhone 6 64GB Factory unlocked new in box on Craigslist. The pictures and details you posted make me feel confident about the quality of these goods. It's rare to find postings as nice as these on Craigslist. I'm absolutely sold on the phone, but I have a maximum budget of $115 to spend. I am willing to meet you at your earliest possible convenience at a location most convenient to you. Thank you again! Looking forward to hearing from you. /  / Best, / Ryan"”
- “Hey friend! Good evening. First of all let me just start up by saying I'm so glad I found your listing since I've been looking for a long time for this phone in this price range. You see, I need it for work and it has been a hassle not being able to complete my work because I don't have this phone. The only downside is that my budget is $115, I know its a lot to ask but is it possible we could reach an agreement? I would deeply appreciate it. Thanks in advance! Have a lovely day.”
- “Greetings! I just found your posting and it has been exactly what I am looking for. I am contacting you to see if it is still available. If so I would like to inquire into purchasing it. The only issue is that I can not afford the asking price of $155. If it is at all possible, would you be open to selling it for $115. I would drive to you so that you do not need to wast gas. Please let me know, thank you for your time.”

The message for Lucky Success (where the ineffective warm strategy succeeds in receiving the discount):

- “Hi there - really great offer for the phone. I have been looking everywhere for this phone and came across your great post. I wanted to buy the for $115 - I promise to take great care of this and am super reliable in getting your money to you efficiently. Please let me know if you can come down to this pricing for this item. WOuld mean the world to me. Thank you~~”

The message for Unlucky Failure (where the effective tough strategy fails in receiving the discount):

- “Hello, I'm interested in your advertisement about the IPHONE 6 Plus 64 GB. I am looking at a couple others listing for the same exact model, also in relatively "almost new" state, but my offer is $115, no more before I take my offer elsewhere. I can assure you that you won't find a higher offer than this an IPHONE 5 plus 64gb in your condition as I've already done my research.”

**Measures:**

*Quantitative learning measure: Message choice (Studies 1 & 2).* “Given what you have learned, if you had to send one of the two messages below, which one would you choose to send? Both messages offer $400 but use different communication styles. Choose the message that will give you the best chance of the seller agreeing to your requested discount.”

- I am interested in buying the used iPhone. However, the asking price is too high for me. Instead, I am offering to pay $400. Does that work? If so, I look forward to doing business with you. If you want to sell your phone, let me know by tomorrow and I can pay in cash when I pick it up. I am flexible on time and place. I look forward to your acceptance of my offer.” (effective communication style)
- Hello! I liked your listing and I am interested in buying the used iPhone. However, the asking price is too high for me even though you clearly took care of it. Instead, would you be willing to accept $400? Does that work? If so, I look forward to doing business with you. If you will be okay with this price, let me know by tomorrow and I can pay in cash when I pick it up. I can meet you at a time and place that is convenient for you. I look forward to your consideration of my offer. Thanks again! (not effective communication style)

*Quantitative learning measure: Direct measure of communication style (Studies 1 & 2)*. “Given what you have learned, what kind of communication style do you think is most effective at getting the discount you want in a negotiation context like this one?” answered on a Likert scale from 1 (Very warm and friendly) to 5 (Very tough and firm), with 3 as mid-point (Neutral).

*Qualitative learning measure: Written negotiation message (Study 1)*. “Given what you have learned about what kind of communication style is effective in getting discounts in this negotiation context, please write a message to the seller offering $400 (not less or more). Remember the phone is listed for $500, but you want a 20% discount, which is $400. Below is the ad for reference.”

*Exploratory measure: Perceived usefulness of learning trials (Studies 1 & 2)*. “From reading the messages that buyers had sent to sellers, how useful do you think they were for learning on how to write an effective message to the seller?” answered on a Likert scale from 1 (Not useful at all) to 5 (A great deal useful).

*Exploratory measure: Confidence (Study 1)*. “How **confident** are you that the message you wrote can receive a discount from the seller?” answered on a Likert scale from 1 (Not at all confident) to (Extremely confident).

*Exploratory measure: Confidence (Study 2)*. “How **confident** are you that you can now write an effective message and receive a discount from the seller?” answered on a Likert scale from 1 (Not at all confident) to (Extremely confident).
