# Supplementary material for: Learning in a noisy world: How lucky successes and unlucky failures shape learning consequences
Source: PLoS One. 2026 Jun 29;21(6):e0352416. doi: 10.1371/journal.pone.0352416 (PMC13313345; doi:10.1371/journal.pone.0352416)
Supplement: Attachment — Submitted filename: Response to Reviewers.docx [file pone.0352416.s003.docx]

Thank you to the review team for their expertise and guidance. Below is our point-by-point response to all comments. For clarity, reviewer comments are presented in bold, while our responses appear in regular text.

**Reviewer #1: the authors offer a nice insight as to how the fundamental attribution error impacts learning processes. it would be useful to have a concluding discussion rather than ending the manuscript abruptly with the ﬁnal experiment.**

Thank you for the kind words on the project. We agree that the manuscript would benefit from a concluding discussion. We have now added a closing paragraph that summarizes the core contribution of the proposed research and discusses its practical implications. Specifically, we highlight that the problem of learning in noisy environments is not simply that feedback is unreliable, but that people are systematically more vulnerable to certain types of noise than others, and that understanding this asymmetry has broad implications for a variety of learning contexts in which people rely on performance feedback to guide behavior. The concluding discussion now reads as follows on pages 14-15:

The proposed research examines a fundamental but underexplored aspect of human learning, namely the ability to recognize and adjust for noise in performance outcomes. By investigating how lucky success and unlucky failure differentially disrupt the learning process, we aim to shed light on why people sometimes do not learn effectively from experience even when feedback is available. Our expected results would suggest that the problem is not simply that learning is noisy, but that people are systematically more vulnerable to certain types of noise than others. Understanding this asymmetry has broad implications for a variety of learning contexts in which people rely on performance feedback to guide their future behavior, and points to the importance of considering not just whether performance feedback is available, but how people are likely to interpret and act on it.

Our paper contributes to research in three ways. First, we contribute to literature on experiential learning by examining the effect of randomness in the process of learning. Experiential learning is powerful, but less controlled than information-based or classroom learning. As individuals try approaches and observe outcomes, they must infer, rather than being explicitly told, what strategies are effective. In this way, experiential learning in real-world settings introduces noise into the learning process. Our work investigates whether and how that noise impacts learning trajectories. Second, we integrate work on decision-making with learning by showing the impact of the fundamental attribution error on experiential learning. Even though failure is often more information rich than success, people are more likely to dismiss the failure as unlucky and assume the success as indicative of their learning about effective strategies. Third, we are the first paper we know of to examine the role of the timing of inaccurate feedback in experiential learning. If it is simply noise that is problematic because people update their beliefs even in the face of inaccurate feedback, then the timing of those outcomes should not matter. However, if our results demonstrate that early lucky success is particularly detrimental to learning, this means that learners anchor on success and are more subject to confirmation bias after success than failure. In other words, they are more likely to interpret subsequent outcomes in light of the early success. This is mitigated if the lucky success occurs later because learners have already processed more information that contradicts the outcome.

From a practical standpoint, our expected results suggest that people can improve learning by responding more skeptically to successful outcomes. If the tendency is to believe them outright, organizations can introduce procedures for pausing and testing its diagnostic value against additional feedback rather than automatically (and potentially erroneously) assuming effective strategies have been implemented. In conclusion, our paper would thus propose that learners would benefit by exercising caution when it comes to early success and reframe “beginner’s luck” as a “novice’s error.”

**Reviewer #2: I really enjoyed reading the manuscript ‘Learning in a noisy world: how lucky successes and unlucky failures shape learning consequences.’ I particularly enjoyed how the study is presented in a straightforward manner and provides an original angle on learning from successes and failures by differentiating by the effect of noise on success and failure. Personally, I study learning from successes and failures in a motor learning context which has shaped my remarks to the study. I have a few major concerns with the study rationale and methods that should be addressed before the manuscript is suitable for publication**

1) **lack of deﬁnition of central concepts**

**Two central concepts haven’t been deﬁned properly: learning and information. Learning seems to be implicitly deﬁned as the changing of behavior. In this case, noisy behavior would be interpreted as learning. To dissociate between changes resulting from noise and changes resulting from learning, the maintenance of behavior should be included in the deﬁnition of learning. Information might be deﬁned as Shannon Entropy, in which deviations from what is expected provide information. When asking participants in pilot B ‘how informative the message was in learning’ they authors seem to interpret information as evidence on what results in success. Providing clear deﬁnitions at the start of the manuscript would help prevent conceptual confusion. In the interpretation of pilot B, the authors should discuss however how the participants might have interpreted the statement that ‘the message was informative in learning.’ The measurement of learning in the planned studies might be updated based on the deﬁnition of learning.**

Thank you for your interest and positive support of our research project. We are grateful for your expertise on this topic. To begin, thank you for pointing out the important observation related to our central concepts. We absolutely agree that the absence of explicit definitions created conceptual ambiguity in the prior draft of the manuscript and we have made three revisions to address this critical concern.

First, we have added a definition to the introduction of what we refer to as “learning” in this research context. Specifically, we rely on a definition of learning in line with experiential learning theory, reinforcement learning, and after-event reviews research as “a process of accurate belief updating that leads to behavioral change, specifically, revising one's beliefs about effective strategies based on performance feedback and adjusting behavior accordingly” (p. 3). In this way, the definition distinguishes genuine learning from noise-driven behavioral change. Under this definition, we would therefore argue that an individual who abandons an effective strategy after an unlucky failure, or who persists with an ineffective strategy after a lucky success, has not effectively learned.

Second, we enhanced the discussion for Pilot B results to address your wise concern about how participants may have interpreted the informativeness measure in alternative ways. We now acknowledge that the phrasing “how informative the message was in learning how to communicate effectively” could have been interpreted differently from its intended meaning of diagnostic value, such as deviation from what was expected or how much uncertainty it reduced, which aligns with a Shannon entropy interpretation. We also clarify that our evidential interpretation is more directly supported by the second measure, participants’ certainty that they had identified why the message succeeded or failed, which more directly captures attribution of causal effectiveness rather than uncertainty reduction. Both interpretations yield the same empirical prediction that success is perceived as more useful than failure, consistent with our pilot data.

Third, in light of the potential alternative ways in which the informativeness measure can be interpreted, we have decided to rely on the term “useful” in our proposed studies. We define the “useful” nature of a performance outcome in the introduction as “the degree to which it serves as a reliable signal of strategy effectiveness” (p. 4). We adopt this definition because our research question concerns how much an outcome instructs the learner about the effectiveness of their strategy, rather than the degree to which an outcome reduces uncertainty. We acknowledge that these definitions are related but distinct, and that the Shannon entropy framework would predict that any surprising outcome, whether success or failure, carries high information. Our definition, by contrast, ties usefulness to the accuracy of the causal inference, which we rely on as the critical construct for learning effective behavior. The relevant wording changes to the measures have been updated in the Supplement.

2) **Lack of justiﬁcation for recruiting large sample sizes**

**In the planned studies, the authors plan to test 800 participants. Such a large sample size creates a risk of type 1 errors because standard errors become very small. The authors should justify based on a power analysis why they need such a large sample size.**

Thank you for pointing out this valid and important concern. We have now added a power analysis to the Methods section related to the proposed studies. We also mention that we aim to follow the convention of recruiting 150 to 200 participants per condition for between-subjects behavioral research (Simmons et al., 2011), resulting in a target sample of 800 participants across four conditions (200 per condition), especially as one of the core dependent variables measuring learning consists of categorical data (i.e., selecting the effective negotiation message). A power analysis using a Chi-square test of independence with four conditions, $\alpha$= .05, and a small-to-medium effect size (*w* = .15) indicates that N = 800 provides over 90% power to detect the predicted effect, and should retain adequate power (greater than 80%) even after anticipated exclusions due to failed attention checks or prior study participation.

3) **Analysis of pilot A**

**I am a bit worried about the analysis of pilot A. The authors mention that average occurrence beliefs were identical between success (32%) and failure (33%) but do not provide information on the statistical test used. If the authors used a two-sample t-test this indeed does not capture within-person differences between the likelihood of lucky success and unlucky failure. However, if the authors used a paired-samples t-test, this does provide information on within-person differences. There is no need to transform the data. I think the analysis on categories rather than continuous data should be replaced with a paired-samples t-test on the continuous data**

Thank you for this observation. We confirm that the statistical test we used was indeed a paired-samples t-test rather than a two-sample t-test, and that these results therefore properly accounted for the within-person nature of the measure. This was not clearly communicated in the original manuscript, and we apologize for this oversight. We have revised the Pilot A results section to explicitly name the test and report the full statistics. We have retained the within-person categorization analysis as a complement to the paired t-test, as it provides additional descriptive insight into the potential skewed distribution of individual-level beliefs that the t-test alone does not convey. We have provided further clarity on the importance of this additional analysis and also included a figure as a visual aid.

4) **lack of embedding in the reinforcement learning literature**

**Reinforcement learning theory [1] is not described but provides an important additional theoretical perspective to the study. In reinforcement learning, humans learn by repeating successes (learning) and adding noise following failure (exploration). In some models applied to reward-based motor learning, the learning and exploration scale with prediction errors: the difference between the current success and the estimated success rate [2]. These ‘rewardbased motor learning’ models are theoretically relevant for two reasons. First, most models do not take into account performance attribution: successful performance is repeated and failed performance is changed [3-5]. Second, the concept of prediction errors might provide an alternative explanation to differences between early and late lucky success or unlucky failure. You are free to decide whether the results are relevant to your work, but I have shown that providing success feedback on large percentage of trials interferes with motor learning [6]. This result seems consistent with your hypothesis that lucky success interferes with learning.**

Thank you for your insight and valuable suggestion. We agree that reinforcement learning theory provides an important complementary perspective that strengthens the theoretical grounding of our work. We view it as very much in line with, and as a subset of experiential learning theory that is shaped by outcome-based feedback. We have now incorporated reinforcement learning theory into the introduction in two ways.

First, we have added reinforcement learning as a complementary theoretical framework alongside experiential learning and after-event reviews research in the first two paragraphs of the introduction. We note that both frameworks converge on the same core assumption, namely that performance outcomes are true and consistent signals of behavioral effectiveness, and that this assumption breaks down in a noisy world. Specifically, the revised paragraph now reads: “Reinforcement learning theory would suggest that people learn by repeating behaviors that led to success and revising behaviors that led to failure, updating their beliefs in proportion to the discrepancy between expected and actual outcomes ). Consistent with this approach, research on after-event reviews describes how people investigate past experiences to determine what behaviors led to subsequent outcomes, as a way to improve future performance [6,10–14]. Both frameworks assume true and consistent causal relationships exist between behaviors and outcomes [6,14]. This assumption, however, breaks down in a noisy world, in which lucky success and unlucky failure sever the causal link between behavior and outcome, rendering feedback to be potentially misleading” (p. 3).

Second, we have engaged with your valuable point about prediction errors as an alternative explanation for our H2 timing prediction. We acknowledge that prediction error models would similarly predict that early noisy signals are more disruptive than late ones, as early outcomes generate larger belief updates when outcome expectations are still uncertain. Our attribution account additionally makes a more specific prediction that cannot be fully explained by prediction error models, which is that the disruptive effect of timing should be asymmetric across outcome valence, such that timing matters more for lucky success than for unlucky failure. We also note with interest your research finding that providing success feedback on a large percentage of trials interferes with motor learning (van der Kooij et al., 2026), which is indeed consistent with our hypothesis (as you have pointed out), and we have now cited this work accordingly. Thank you for bringing that important work to our attention. Study 2 is designed to test the prediction of the asymmetric effect of timing across outcome valence, which would provide support for our attribution account beyond what a purely prediction-error-based account would predict.

Thank you again for the opportunity to revise our manuscript. We look forward to hearing from you.
