# Supplementary material for: Learning in a noisy world: How lucky successes and unlucky failures shape learning consequences
Source: PLoS One. 2026 Jun 29;21(6):e0352416. doi: 10.1371/journal.pone.0352416 (PMC13313345; doi:10.1371/journal.pone.0352416)
Supplement: Attachment — Submitted filename: Response to Reviewers_6.09.26.docx [file pone.0352416.s004.docx]

Thank you again to the review team. Below is our point-by-point response to all comments. Reviewer comments are presented in bold, while our responses appear in regular text.

**Additional Editor Comments: I read the revised manuscript with great pleasure and believe that it is almost ready for publication. Only Reviewer 1 has raised one remaining minor concern regarding the way the manuscript describes random noise as potentially disrupting reinforcement learning. Please address this comment in your revision by tempering your statement. I hope that you will be able to address this final minor concern, as I believe doing so will further strengthen the manuscript and its contribution to the journal. Provided that this concern is satisfactorily addressed, I anticipate being able to reach a positive final decision promptly.**

Thank you for the encouragement - we are excited for the opportunity to finalize our manuscript. We have addressed Reviewer 1’s important concern. We absolutely agree that tempering our language regarding how random noise can potentially disrupt learning will further strengthen our manuscript. Specifically, we have made changes to the sentence Reviewer 1 pointed out (page 3), and also identified 3 other instances (pages 4, 8, and 14) where we believed tempering our language would be similarly helpful. The exact changes we made are detailed below.

We also corrected two citation formatting typos (pages 3 & 11). We also replaced “500 hits” with “500 previous submissions” (pages 8 & 12) as the former terminology is used by Amazon’s Mechnical Turk and the latter terminology is used by the Prolific platform, and we plan to collect our data on Prolific.

**Reviewer #1: A nice revision. My only concern at this point is that at one or two points you characterize reinforcement learning as "breaking down" under a setting of noise. Yes, it can become problematic in settings with a high level of noise, but the vast literature on bandit models and such illustrate that learning can still be effective even in a noisy world. Again, I suggest offer somewhat more tempered language than "breaking down".**

Thank you for bringing our attention to this important suggestion. We have made the following changes to temper our language in this revision.

The sentence you identified (page 3) previously read as, “This assumption, however, breaks down in a noisy world, in which lucky success and unlucky failure sever the causal link between behavior and outcome, rendering feedback to be potentially misleading.”

It now reads (with the new language highlighted for easier review), “This assumption, however, starts to weaken in a noisy world, in which lucky success and unlucky failure can obscure the causal link between behavior and outcome, resulting in feedback that can be potentially misleading.”

Additionally, we have gone through the manuscript and made similar edits to temper our language in 3 other instances. We replaced “disrupt learning” with “affect learning” on page 4; we replaced “disturb learning” with “impair learning” on page 8; and we replaced “disrupt learning” with “interfere with learning” on page 14.

We believe this revised language better captures the phenomenon, and we are grateful for your suggestion.

**Reviewer #2: Thank you for considering my comments to the previous version and providing a clear and detailed response. All my concerns have been appropriately addressed and I look forward to reading the outcomes of the planned study. Best regards, Katinka**

Thank you for your kind words on the project. We are excited to move the project forward!
