# Supplementary material for: Learning in a noisy world: How lucky successes and unlucky failures shape learning consequences
Source: PLoS One. 2026 Jun 29;21(6):e0352416. doi: 10.1371/journal.pone.0352416 (PMC13313345; doi:10.1371/journal.pone.0352416)
Supplement: Peer review history

# Peer review: Decision Letter 0

**DOI:** 10.1371/journal.pone.0352416.r001

**Authors:** Annesha Sil

29 Mar 2026

PONE-D-26-04456

Learning in a Noisy World: How lucky successes and unlucky failures shape learning consequences

PLOS One

Dear Dr. Jeong,

Thank you for submitting your manuscript to PLOS ONE. After careful consideration, we feel that it has merit but does not fully meet PLOS ONE’s publication criteria as it currently stands. Therefore, we invite you to submit a revised version of the manuscript that addresses the points raised during the review process.

The reviewers have requested some further clarifications regarding the methodology and analysis. Could you please revise your manuscript to address each of their comments?

Please submit your revised manuscript by May 11 2026 11:59PM. If you will need more time than this to complete your revisions, please reply to this message or contact the journal office at plosone@plos.org. When you're ready to submit your revision, log on to https://www.editorialmanager.com/pone/ and select the 'Submissions Needing Revision' folder to locate your manuscript file.

Please include the following items when submitting your revised manuscript:

- A letter that responds to each point raised by the academic editor and reviewer(s). You should upload this letter as a separate file labeled 'Response to Reviewers'.
- A marked-up copy of your manuscript that highlights changes made to the original version. You should upload this as a separate file labeled 'Revised Manuscript with Track Changes'.
- An unmarked version of your revised paper without tracked changes. You should upload this as a separate file labeled 'Manuscript'.

If applicable, we recommend that you deposit your laboratory protocols in protocols.io to enhance the reproducibility of your results. Protocols.io assigns your protocol its own identifier (DOI) so that it can be cited independently in the future. For instructions see: https://journals.plos.org/plosone/s/submission-guidelines#loc-laboratory-protocols. Additionally, PLOS ONE offers an option for publishing peer-reviewed Lab Protocol articles, which describe protocols hosted on protocols.io. Read more information on sharing protocols at https://plos.org/protocols?utm_medium=editorial-email&utm_source=authorletters&utm_campaign=protocols.

As the corresponding author, your ORCID iD is verified in the submission system and will appear in the published article. PLOS supports the use of ORCID, and we encourage all coauthors to register for an ORCID iD and use it as well. Please encourage your coauthors to verify their ORCID iD within the submission system before final acceptance, as unverified ORCID iDs will not appear in the published article. *Only*  the individual author can complete the verification step; PLOS staff *cannot*  verify ORCID iDs on behalf of authors.

We look forward to receiving your revised manuscript.

Kind regards,

Annesha Sil, Ph.D.

Staff Editor

PLOS One

Journal Requirements:

When submitting your revision, we need you to address these additional requirements.

1. Please ensure that your manuscript meets PLOS ONE's style requirements, including those for file naming. The PLOS ONE style templates can be found at

https://journals.plos.org/plosone/s/file?id=wjVg/PLOSOne_formatting_sample_main_body.pdf and

https://journals.plos.org/plosone/s/file?id=ba62/PLOSOne_formatting_sample_title_authors_affiliations.pdf

2. In your cover letter, please confirm that the research you have described in your manuscript, including participant recruitment, data collection, modification, or processing, has not started and will not start until after your paper has been accepted to the journal (assuming data need to be collected or participants recruited specifically for your study). In order to proceed with your submission, you must provide confirmation.

3. Your ethics statement should only appear in the Methods section of your manuscript. If your ethics statement is written in any section besides the Methods, please move it to the Methods section and delete it from any other section. Please ensure that your ethics statement is included in your manuscript, as the ethics statement entered into the online submission form will not be published alongside your manuscript.

4. Please include captions for your Supporting Information files at the end of your manuscript, and update any in-text citations to match accordingly. Please see our Supporting Information guidelines for more information: http://journals.plos.org/plosone/s/supporting-information.

5. If the reviewer comments include a recommendation to cite specific previously published works, please review and evaluate these publications to determine whether they are relevant and should be cited. There is no requirement to cite these works unless the editor has indicated otherwise.

[Note: HTML markup is below. Please do not edit.]

Reviewers' comments:

Reviewer's Responses to Questions

**Comments to the Author**

1. Does the manuscript provide a valid rationale for the proposed study, with clearly identified and justified research questions?

The research question outlined is expected to address a valid academic problem or topic and contribute to the base of knowledge in the field.

Reviewer #1: Yes

Reviewer #2: Yes

**********

2. Is the protocol technically sound and planned in a manner that will lead to a meaningful outcome and allow testing the stated hypotheses?

The manuscript should describe the methods in sufficient detail to prevent undisclosed flexibility in the experimental procedure or analysis pipeline, including sufficient outcome-neutral conditions (e.g. necessary controls, absence of floor or ceiling effects) to test the proposed hypotheses and a statistical power analysis where applicable. As there may be aspects of the methodology and analysis which can only be refined once the work is undertaken, authors should outline potential assumptions and explicitly describe what aspects of the proposed analyses, if any, are exploratory.

Reviewer #1: Yes

Reviewer #2: Partly

**********

3. Is the methodology feasible and described in sufficient detail to allow the work to be replicable?

Reviewer #1: Yes

Reviewer #2: Yes

**********

4. Have the authors described where all data underlying the findings will be made available when the study is complete?

The PLOS Data policy requires authors to make all data underlying the findings described in their manuscript fully available without restriction, with rare exception, at the time of publication. The data should be provided as part of the manuscript or its supporting information, or deposited to a public repository. For example, in addition to summary statistics, the data points behind means, medians and variance measures should be available. If there are restrictions on publicly sharing data—e.g. participant privacy or use of data from a third party—those must be specified.

Reviewer #1: Yes

Reviewer #2: No

**********

5. Is the manuscript presented in an intelligible fashion and written in standard English?

*PLOS ONE*  does not copyedit accepted manuscripts, so the language in submitted articles must be clear, correct, and unambiguous. Any typographical or grammatical errors should be corrected at revision, so please note any specific errors here.

Reviewer #1: Yes

Reviewer #2: Yes

**********

6. Review Comments to the Author

Please use the space provided to explain your answers to the questions above and, if applicable, provide comments about issues authors must address before this protocol can be accepted for publication. You may also include additional comments for the author, including concerns about research or publication ethics.

You may also provide optional suggestions and comments to authors that they might find helpful in planning their study.

(Please upload your review as an attachment if it exceeds 20,000 characters)

Reviewer #1: the authors offer a nice insight as to how the fundamental attribution error impacts learning processes. it would be useful to have a concluding discussion rather than ending the manuscript abruptly with the final experiment.

Reviewer #2: I really enjoyed reading the manuscript ‘Learning in a noisy world: how lucky successes and unlucky failures shape learning consequences.’ I particularly enjoyed how the study is presented in a straightforward manner and provides an original angle on learning from successes and failures by differentiating by the effect of noise on success and failure. Personally, I study learning from successes and failures in a motor learning context which has shaped my remarks to the study. I have a few major concerns with the study rationale and methods that should be addressed before the manuscript is suitable for publication

1) lack of definition of central concepts

Two central concepts haven’t been defined properly: learning and information. Learning seems to be implicitly defined as the changing of behavior. In this case, noisy behavior would be interpreted as learning. To dissociate between changes resulting from noise and changes resulting from learning, the maintenance of behavior should be included in the definition of learning. Information might be defined as Shannon Entropy, in which deviations from what is expected provide information. When asking participants in pilot B ‘how informative the message was in learning’ they authors seem to interpret information as evidence on what results in success. Providing clear definitions at the start of the manuscript would help prevent conceptual confusion. In the interpretation of pilot B, the authors should discuss however how the participants might have interpreted the statement that ‘the message was informative in learning.’ The measurement of learning in the planned studies might be updated based on the definition of learning.

2) Lack of justification for recruiting large sample sizes

In the planned studies, the authors plan to test 800 participants. Such a large sample size creates a risk of type 1 errors because standard errors become very small. The authors should justify based on a power analysis why they need such a large sample size.

3) Analysis of pilot A

I am a bit worried about the analysis of pilot A. The authors mention that average occurrence beliefs were identical between success (32%) and failure (33%) but do not provide information on the statistical test used. If the authors used a two-sample t-test this indeed does not capture within-person differences between the likelihood of lucky success and unlucky failure. However, if the authors used a paired-samples t-test, this does provide information on within-person differences. There is no need to transform the data. I think the analysis on categories rather than continuous data should be replaced with a paired-samples t-test on the continuous data

4) lack of embedding in the reinforcement learning literature

Reinforcement learning theory [1] is not described but provides an important additional theoretical perspective to the study. In reinforcement learning, humans learn by repeating successes (learning) and adding noise following failure (exploration). In some models applied to reward-based motor learning, the learning and exploration scale with prediction errors: the difference between the current success and the estimated success rate [2]. These ‘reward-based motor learning’ models are theoretically relevant for two reasons. First, most models do not take into account performance attribution: successful performance is repeated and failed performance is changed [3-5]. Second, the concept of prediction errors might provide an alternative explanation to differences between early and late lucky success or unlucky failure. You are free to decide whether the results are relevant to your work, but I have shown that providing success feedback on large percentage of trials interferes with motor learning [6]. This result seems consistent with your hypothesis that lucky success interferes with learning.

1. Sutton, R.S. and A.G. Barto, Reinforcement learning. Adaptive computation and machine learning series. 2018, Cambridge, MA: The MIT Press.

2. Dhawale, A.K., et al., Adaptive regulation of motor variability. Current Biology, 2019. 29(21): p. 3551-3562.

3. Therrien, A.S., D.M. Wolpert, and A.J. Bastian, Increasing motor noise impairs reinforcement learning in healthy individuals. eNeuro, 2018. 5(3): p. e0050-18.2018 1–14.

4. Roth, A.M., et al., Reinforcement-based processes actively regulate motor exploration along redundant solution manifolds. Proceedings of the royal society B, 2023. 290: p. 20231475.

5. Izawa, J. and R. Shadmehr, Learning from sensory and reward prediction errors during motor adaptation. PLOS computational biology, 2011. 7(3): p. e1002012.

6. van der Kooij, K., et al., Enforcing a high success percentage interferes with reward-based motor learning. Scientific Reports, 2026.

**********

7. PLOS authors have the option to publish the peer review history of their article (what does this mean?). If published, this will include your full peer review and any attached files.

If you choose “no”, your identity will remain anonymous but your review may still be made public.

**Do you want your identity to be public for this peer review?**  For information about this choice, including consent withdrawal, please see our Privacy Policy.

Reviewer #1: No

Reviewer #2: No

**********

[NOTE: If reviewer comments were submitted as an attachment file, they will be attached to this email and accessible via the submission site. Please log into your account, locate the manuscript record, and check for the action link "View Attachments". If this link does not appear, there are no attachment files.]

To ensure your figures meet our technical requirements, please review our figure guidelines: https://journals.plos.org/plosone/s/figures

You may also use PLOS’s free figure tool, NAAS, to help you prepare publication quality figures: https://journals.plos.org/plosone/s/figures#loc-tools-for-figure-preparation.

NAAS will assess whether your figures meet our technical requirements by comparing each figure against our figure specifications.

# Author comment: Author response to Decision Letter 1

**DOI:** 10.1371/journal.pone.0352416.r002

18 May 2026

Thank you to the review team for their expertise and guidance. Below is our point-by-point response to all comments. For clarity, reviewer comments are presented in bold, while our responses appear in regular text.

Reviewer #1: the authors offer a nice insight as to how the fundamental attribution error impacts learning processes. it would be useful to have a concluding discussion rather than ending the manuscript abruptly with the ﬁnal experiment.

Thank you for the kind words on the project. We agree that the manuscript would benefit from a concluding discussion. We have now added a closing paragraph that summarizes the core contribution of the proposed research and discusses its practical implications. Specifically, we highlight that the problem of learning in noisy environments is not simply that feedback is unreliable, but that people are systematically more vulnerable to certain types of noise than others, and that understanding this asymmetry has broad implications for a variety of learning contexts in which people rely on performance feedback to guide behavior. The concluding discussion now reads as follows on pages 14-15:

The proposed research examines a fundamental but underexplored aspect of human learning, namely the ability to recognize and adjust for noise in performance outcomes. By investigating how lucky success and unlucky failure differentially disrupt the learning process, we aim to shed light on why people sometimes do not learn effectively from experience even when feedback is available. Our expected results would suggest that the problem is not simply that learning is noisy, but that people are systematically more vulnerable to certain types of noise than others. Understanding this asymmetry has broad implications for a variety of learning contexts in which people rely on performance feedback to guide their future behavior, and points to the importance of considering not just whether performance feedback is available, but how people are likely to interpret and act on it.

Our paper contributes to research in three ways. First, we contribute to literature on experiential learning by examining the effect of randomness in the process of learning. Experiential learning is powerful, but less controlled than information-based or classroom learning. As individuals try approaches and observe outcomes, they must infer, rather than being explicitly told, what strategies are effective. In this way, experiential learning in real-world settings introduces noise into the learning process. Our work investigates whether and how that noise impacts learning trajectories. Second, we integrate work on decision-making with learning by showing the impact of the fundamental attribution error on experiential learning. Even though failure is often more information rich than success, people are more likely to dismiss the failure as unlucky and assume the success as indicative of their learning about effective strategies. Third, we are the first paper we know of to examine the role of the timing of inaccurate feedback in experiential learning. If it is simply noise that is problematic because people update their beliefs even in the face of inaccurate feedback, then the timing of those outcomes should not matter. However, if our results demonstrate that early lucky success is particularly detrimental to learning, this means that learners anchor on success and are more subject to confirmation bias after success than failure. In other words, they are more likely to interpret subsequent outcomes in light of the early success. This is mitigated if the lucky success occurs later because learners have already processed more information that contradicts the outcome.

From a practical standpoint, our expected results suggest that people can improve learning by responding more skeptically to successful outcomes. If the tendency is to believe them outright, organizations can introduce procedures for pausing and testing its diagnostic value against additional feedback rather than automatically (and potentially erroneously) assuming effective strategies have been implemented. In conclusion, our paper would thus propose that learners would benefit by exercising caution when it comes to early success and reframe “beginner’s luck” as a “novice’s error.”

Reviewer #2: I really enjoyed reading the manuscript ‘Learning in a noisy world: how lucky successes and unlucky failures shape learning consequences.’ I particularly enjoyed how the study is presented in a straightforward manner and provides an original angle on learning from successes and failures by differentiating by the effect of noise on success and failure. Personally, I study learning from successes and failures in a motor learning context which has shaped my remarks to the study. I have a few major concerns with the study rationale and methods that should be addressed before the manuscript is suitable for publication

lack of deﬁnition of central concepts

Two central concepts haven’t been deﬁned properly: learning and information. Learning seems to be implicitly deﬁned as the changing of behavior. In this case, noisy behavior would be interpreted as learning. To dissociate between changes resulting from noise and changes resulting from learning, the maintenance of behavior should be included in the deﬁnition of learning. Information might be deﬁned as Shannon Entropy, in which deviations from what is expected provide information. When asking participants in pilot B ‘how informative the message was in learning’ they authors seem to interpret information as evidence on what results in success. Providing clear deﬁnitions at the start of the manuscript would help prevent conceptual confusion. In the interpretation of pilot B, the authors should discuss however how the participants might have interpreted the statement that ‘the message was informative in learning.’ The measurement of learning in the planned studies might be updated based on the deﬁnition of learning.

Thank you for your interest and positive support of our research project. We are grateful for your expertise on this topic. To begin, thank you for pointing out the important observation related to our central concepts. We absolutely agree that the absence of explicit definitions created conceptual ambiguity in the prior draft of the manuscript and we have made three revisions to address this critical concern.

First, we have added a definition to the introduction of what we refer to as “learning” in this research context. Specifically, we rely on a definition of learning in line with experiential learning theory, reinforcement learning, and after-event reviews research as “a process of accurate belief updating that leads to behavioral change, specifically, revising one's beliefs about effective strategies based on performance feedback and adjusting behavior accordingly” (p. 3). In this way, the definition distinguishes genuine learning from noise-driven behavioral change. Under this definition, we would therefore argue that an individual who abandons an effective strategy after an unlucky failure, or who persists with an ineffective strategy after a lucky success, has not effectively learned.

Second, we enhanced the discussion for Pilot B results to address your wise concern about how participants may have interpreted the informativeness measure in alternative ways. We now acknowledge that the phrasing “how informative the message was in learning how to communicate effectively” could have been interpreted differently from its intended meaning of diagnostic value, such as deviation from what was expected or how much uncertainty it reduced, which aligns with a Shannon entropy interpretation. We also clarify that our evidential interpretation is more directly supported by the second measure, participants’ certainty that they had identified why the message succeeded or failed, which more directly captures attribution of causal effectiveness rather than uncertainty reduction. Both interpretations yield the same empirical prediction that success is perceived as more useful than failure, consistent with our pilot data.

Third, in light of the potential alternative ways in which the informativeness measure can be interpreted, we have decided to rely on the term “useful” in our proposed studies. We define the “useful” nature of a performance outcome in the introduction as “the degree to which it serves as a reliable signal of strategy effectiveness” (p. 4). We adopt this definition because our research question concerns how much an outcome instructs the learner about the effectiveness of their strategy, rather than the degree to which an outcome reduces uncertainty. We acknowledge that these definitions are related but distinct, and that the Shannon entropy framework would predict that any surprising outcome, whether success or failure, carries high information. Our definition, by contrast, ties usefulness to the accuracy of the causal inference, which we rely on as the critical construct for learning effective behavior. The relevant wording changes to the measures have been updated in the Supplement.

Lack of justiﬁcation for recruiting large sample sizes

In the planned studies, the authors plan to test 800 participants. Such a large sample size creates a risk of type 1 errors because standard errors become very small. The authors should justify based on a power analysis why they need such a large sample size.

Thank you for pointing out this valid and important concern. We have now added a power analysis to the Methods section related to the proposed studies. We also mention that we aim to follow the convention of recruiting 150 to 200 participants per condition for between-subjects behavioral research (Simmons et al., 2011), resulting in a target sample of 800 participants across four conditions (200 per condition), especially as one of the core dependent variables measuring learning consists of categorical data (i.e., selecting the effective negotiation message). A power analysis using a Chi-square test of independence with four conditions, α = .05, and a small-to-medium effect size (w = .15) indicates that N = 800 provides over 90% power to detect the predicted effect, and should retain adequate power (greater than 80%) even after anticipated exclusions due to failed attention checks or prior study participation.

Analysis of pilot A

I am a bit worried about the analysis of pilot A. The authors mention that average occurrence beliefs were identical between success (32%) and failure (33%) but do not provide information on the statistical test used. If the authors used a two-sample t-test this indeed does not capture within-person differences between the likelihood of lucky success and unlucky failure. However, if the authors used a paired-samples t-test, this does provide information on within-person differences. There is no need to transform the data. I think the analysis on categories rather than continuous data should be replaced with a paired-samples t-test on the continuous data

Thank you for this observation. We confirm that the statistical test we used was indeed a paired-samples t-test rather than a two-sample t-test, and that these results therefore properly accounted for the within-person nature of the measure. This was not clearly communicated in the original manuscript, and we apologize for this oversight. We have revised the Pilot A results section to explicitly name the test and report the full statistics. We have retained the within-person categorization analysis as a complement to the paired t-test, as it provides additional descriptive insight into the potential skewed distribution of individual-level beliefs that the t-test alone does not convey. We have provided further clarity on the importance of this additional analysis and also included a figure as a visual aid.

lack of embedding in the reinforcement learning literature

Reinforcement learning theory [1] is not described but provides an important additional theoretical perspective to the study. In reinforcement learning, humans learn by repeating successes (learning) and adding noise following failure (exploration). In some models applied to reward-based motor learning, the learning and exploration scale with prediction errors: the difference between the current success and the estimated success rate [2]. These ‘rewardbased motor learning’ models are theoretically relevant for two reasons. First, most models do not take into account performance attribution: successful performance is repeated and failed performance is changed [3-5]. Second, the concept of prediction errors might provide an alternative explanation to differences between early and late lucky success or unlucky failure. You are free to decide whether the results are relevant to your work, but I have shown that providing success feedback on large percentage of trials interferes with motor learning [6]. This result seems consistent with your hypothesis that lucky success interferes with learning.

Thank you for your insight and valuable suggestion. We agree that reinforcement learning theory provides an important complementary perspective that strengthens the theoretical grounding of our work. We view it as very much in line with, and as a subset of experiential learning theory that is shaped by outcome-based feedback. We have now incorporated reinforcement learning theory into the introduction in two ways.

First, we have added reinforcement learning as a complementary theoretical framework alongside experiential learning and after-event reviews research in the first two paragraphs of the introduction. We note that both frameworks converge on the same core assumption, namely that performance outcomes are true and consistent signals of behavioral effectiveness, and that this assumption breaks down in a noisy world. Specifically, the revised paragraph now reads: “Reinforcement learning theory would suggest that people learn by repeating behaviors that led to success and revising behaviors that led to failure, updating their beliefs in proportion to the discrepancy between expected and actual outcomes ). Consistent with this approach, research on after-event reviews describes how people investigate past experiences to determine what behaviors led to subsequent outcomes, as a way to improve future performance [6,10–14]. Both frameworks assume true and consistent causal relationships exist between behaviors and outcomes [6,14]. This assumption, however, breaks down in a noisy world, in which lucky success and unlucky failure sever the causal link between behavior and outcome, rendering feedback to be potentially misleading” (p. 3).

Second, we have engaged with your valuable point about prediction errors as an alternative explanation for our H2 timing prediction. We acknowledge that prediction error models would similarly predict that early noisy signals are more disruptive than late ones, as early outcomes generate larger belief updates when outcome expectations are still uncertain. Our attribution account additionally makes a more specific prediction that cannot be fully explained by prediction error models, which is that the disruptive effect of timing should be asymmetric across outcome valence, such that timing matters more for lucky success than for unlucky failure. We also note with interest your research finding that providing success feedback on a large percentage of trials interferes with motor learning (van der Kooij et al., 2026), which is indeed consistent with our hypothesis (as you have pointed out), and we have now cited this work accordingly. Thank you for bringing that important work to our attention. Study 2 is designed to test the prediction of the asymmetric effect of timing across outcome valence, which would provide support for our attribution account beyond what a purely prediction-error-based account would predict.

Thank you again for the opportunity to revise our manuscript. We look forward to hearing from you.

Attachment Submitted filename: Response to Reviewers.docx

# Peer review: Decision Letter 1

**DOI:** 10.1371/journal.pone.0352416.r003

**Authors:** Nicola Vasta

8 Jun 2026

PONE-D-26-04456R1

Learning in a Noisy World: How lucky successes and unlucky failures shape learning consequences

PLOS One

Dear Dr. Jeong,

Thank you for submitting your manuscript to PLOS ONE. After careful consideration, we feel that it has merit but does not fully meet PLOS ONE’s publication criteria as it currently stands. Therefore, we invite you to submit a revised version of the manuscript that addresses the points raised during the review process.

Please submit your revised manuscript by Jul 23 2026 11:59PM. If you will need more time than this to complete your revisions, please reply to this message or contact the journal office at plosone@plos.org. When you're ready to submit your revision, log on to https://www.editorialmanager.com/pone/ and select the 'Submissions Needing Revision' folder to locate your manuscript file.

Please include the following items when submitting your revised manuscript:

- A letter that responds to each point raised by the academic editor and reviewer(s). You should upload this letter as a separate file labeled 'Response to Reviewers'.
- A marked-up copy of your manuscript that highlights changes made to the original version. You should upload this as a separate file labeled 'Revised Manuscript with Track Changes'.
- An unmarked version of your revised paper without tracked changes. You should upload this as a separate file labeled 'Manuscript'.

If you would like to make changes to your financial disclosure, please include your updated statement in your cover letter. Guidelines for resubmitting your figure files are available below the reviewer comments at the end of this letter.

If applicable, we recommend that you deposit your laboratory protocols in protocols.io to enhance the reproducibility of your results. Protocols.io assigns your protocol its own identifier (DOI) so that it can be cited independently in the future. For instructions see: https://journals.plos.org/plosone/s/submission-guidelines#loc-laboratory-protocols. Additionally, PLOS ONE offers an option for publishing peer-reviewed Lab Protocol articles, which describe protocols hosted on protocols.io. Read more information on sharing protocols at https://plos.org/protocols?utm_medium=editorial-email&utm_source=authorletters&utm_campaign=protocols.

As the corresponding author, your ORCID iD is verified in the submission system and will appear in the published article. PLOS supports the use of ORCID, and we encourage all coauthors to register for an ORCID iD and use it as well. Please encourage your coauthors to verify their ORCID iD within the submission system before final acceptance, as unverified ORCID iDs will not appear in the published article. *Only*  the individual author can complete the verification step; PLOS staff *cannot*  verify ORCID iDs on behalf of authors.

We look forward to receiving your revised manuscript.

Kind regards,

Nicola Vasta

Academic Editor

PLOS One

Journal Requirements:

If the reviewer comments include a recommendation to cite specific previously published works, please review and evaluate these publications to determine whether they are relevant and should be cited. There is no requirement to cite these works unless the editor has indicated otherwise.

Please review your reference list to ensure that it is complete and correct. If you have cited papers that have been retracted, please include the rationale for doing so in the manuscript text, or remove these references and replace them with relevant current references. Any changes to the reference list should be mentioned in the rebuttal letter that accompanies your revised manuscript. If you need to cite a retracted article, indicate the article’s retracted status in the References list and also include a citation and full reference for the retraction notice.

**Additional Editor Comments:**

I read the revised manuscript with great pleasure and believe that it is almost ready for publication. Only Reviewer 1 has raised one remaining minor concern regarding the way the manuscript describes random noise as potentially disrupting reinforcement learning. Please address this comment in your revision by tempering your statement.

I hope that you will be able to address this final minor concern, as I believe doing so will further strengthen the manuscript and its contribution to the journal. Provided that this concern is satisfactorily addressed, I anticipate being able to reach a positive final decision promptly.

The reviewers' comments are appended below my signature.

[Note: HTML markup is below. Please do not edit.]

Reviewers' comments:

Reviewer's Responses to Questions

**Comments to the Author**

1. Does the manuscript provide a valid rationale for the proposed study, with clearly identified and justified research questions?

The research question outlined is expected to address a valid academic problem or topic and contribute to the base of knowledge in the field.

Reviewer #1: Yes

Reviewer #2: Yes

**********

2. Is the protocol technically sound and planned in a manner that will lead to a meaningful outcome and allow testing the stated hypotheses?

The manuscript should describe the methods in sufficient detail to prevent undisclosed flexibility in the experimental procedure or analysis pipeline, including sufficient outcome-neutral conditions (e.g. necessary controls, absence of floor or ceiling effects) to test the proposed hypotheses and a statistical power analysis where applicable. As there may be aspects of the methodology and analysis which can only be refined once the work is undertaken, authors should outline potential assumptions and explicitly describe what aspects of the proposed analyses, if any, are exploratory.

Reviewer #1: Yes

Reviewer #2: Yes

**********

3. Is the methodology feasible and described in sufficient detail to allow the work to be replicable?

Reviewer #1: Yes

Reviewer #2: Yes

**********

4. Have the authors described where all data underlying the findings will be made available when the study is complete?

The PLOS Data policy requires authors to make all data underlying the findings described in their manuscript fully available without restriction, with rare exception, at the time of publication. The data should be provided as part of the manuscript or its supporting information, or deposited to a public repository. For example, in addition to summary statistics, the data points behind means, medians and variance measures should be available. If there are restrictions on publicly sharing data—e.g. participant privacy or use of data from a third party—those must be specified.

Reviewer #1: Yes

Reviewer #2: Yes

**********

5. Is the manuscript presented in an intelligible fashion and written in standard English?

*PLOS ONE*  does not copyedit accepted manuscripts, so the language in submitted articles must be clear, correct, and unambiguous. Any typographical or grammatical errors should be corrected at revision, so please note any specific errors here.

Reviewer #1: Yes

Reviewer #2: Yes

**********

6. Review Comments to the Author

Please use the space provided to explain your answers to the questions above and, if applicable, provide comments about issues authors must address before this protocol can be accepted for publication. You may also include additional comments for the author, including concerns about research or publication ethics.

You may also provide optional suggestions and comments to authors that they might find helpful in planning their study.

(Please upload your review as an attachment if it exceeds 20,000 characters)

Reviewer #1: A nice revision. My only concern at this point is that at one or two points you characterize reinforcement learning as "breaking down" under a setting of noise. Yes, it can become problematic in settings with a high level of noise, but the vast literature on bandit models and such illustrate that learning can still be effective even in a noisy world. Again, I suggest offer somewhat more tempered language than "breaking down".

Reviewer #2: Thank you for considering my comments to the previous version and providing a clear and detailed response. All my concerns have been appropriately addressed and I look forward to reading the outcomes of the planned study. Best regards, Katinka

**********

7. PLOS authors have the option to publish the peer review history of their article (what does this mean?). If published, this will include your full peer review and any attached files.

If you choose “no”, your identity will remain anonymous but your review may still be made public.

**Do you want your identity to be public for this peer review?**  For information about this choice, including consent withdrawal, please see our Privacy Policy.

Reviewer #1: No

Reviewer #2: **Yes:** Katinka van der Kooij

**********

[NOTE: If reviewer comments were submitted as an attachment file, they will be attached to this email and accessible via the submission site. Please log into your account, locate the manuscript record, and check for the action link "View Attachments". If this link does not appear, there are no attachment files.]

To ensure your figures meet our technical requirements, please review our figure guidelines: https://journals.plos.org/plosone/s/figures

You may also use PLOS’s free figure tool, NAAS, to help you prepare publication quality figures: https://journals.plos.org/plosone/s/figures#loc-tools-for-figure-preparation.

NAAS will assess whether your figures meet our technical requirements by comparing each figure against our figure specifications.

# Author comment: Author response to Decision Letter 2

**DOI:** 10.1371/journal.pone.0352416.r004

8 Jun 2026

Thank you again to the review team. Below is our point-by-point response to all comments. Reviewer comments are presented in bold, while our responses appear in regular text.

Additional Editor Comments: I read the revised manuscript with great pleasure and believe that it is almost ready for publication. Only Reviewer 1 has raised one remaining minor concern regarding the way the manuscript describes random noise as potentially disrupting reinforcement learning. Please address this comment in your revision by tempering your statement. I hope that you will be able to address this final minor concern, as I believe doing so will further strengthen the manuscript and its contribution to the journal. Provided that this concern is satisfactorily addressed, I anticipate being able to reach a positive final decision promptly.

Thank you for the encouragement - we are excited for the opportunity to finalize our manuscript. We have addressed Reviewer 1’s important concern. We absolutely agree that tempering our language regarding how random noise can potentially disrupt learning will further strengthen our manuscript. Specifically, we have made changes to the sentence Reviewer 1 pointed out (page 3), and also identified 3 other instances (pages 4, 8, and 14) where we believed tempering our language would be similarly helpful. The exact changes we made are detailed below.

We also corrected two citation formatting typos (pages 3 & 11). We also replaced “500 hits” with “500 previous submissions” (pages 8 & 12) as the former terminology is used by Amazon’s Mechnical Turk and the latter terminology is used by the Prolific platform, and we plan to collect our data on Prolific.

Reviewer #1: A nice revision. My only concern at this point is that at one or two points you characterize reinforcement learning as "breaking down" under a setting of noise. Yes, it can become problematic in settings with a high level of noise, but the vast literature on bandit models and such illustrate that learning can still be effective even in a noisy world. Again, I suggest offer somewhat more tempered language than "breaking down".

Thank you for bringing our attention to this important suggestion. We have made the following changes to temper our language in this revision.

The sentence you identified (page 3) previously read as, “This assumption, however, breaks down in a noisy world, in which lucky success and unlucky failure sever the causal link between behavior and outcome, rendering feedback to be potentially misleading.”

It now reads (with the new language highlighted for easier review), “This assumption, however, starts to weaken in a noisy world, in which lucky success and unlucky failure can obscure the causal link between behavior and outcome, resulting in feedback that can be potentially misleading.”

Additionally, we have gone through the manuscript and made similar edits to temper our language in 3 other instances. We replaced “disrupt learning” with “affect learning” on page 4; we replaced “disturb learning” with “impair learning” on page 8; and we replaced “disrupt learning” with “interfere with learning” on page 14.

We believe this revised language better captures the phenomenon, and we are grateful for your suggestion.

Reviewer #2: Thank you for considering my comments to the previous version and providing a clear and detailed response. All my concerns have been appropriately addressed and I look forward to reading the outcomes of the planned study. Best regards, Katinka

Thank you for your kind words on the project. We are excited to move the project forward!

Attachment Submitted filename: Response to Reviewers_6.09.26.docx

# Editor report: Decision Letter 2

**DOI:** 10.1371/journal.pone.0352416.r005

**Authors:** Nicola Vasta

10 Jun 2026

Learning in a Noisy World: How lucky successes and unlucky failures shape learning consequences

PONE-D-26-04456R2

Dear Dr. Jeong,

We’re pleased to inform you that your manuscript has been judged scientifically suitable for publication and will be formally accepted for publication once it meets all outstanding technical requirements.

Within one week, you’ll receive an e-mail detailing the required amendments. When these have been addressed, you’ll receive a formal acceptance letter and your manuscript will be scheduled for publication.

An invoice will be generated when your article is formally accepted. Please note, if your institution has a publishing partnership with PLOS and your article meets the relevant criteria, all or part of your publication costs will be covered. Please make sure your user information is up-to-date by logging into Editorial Manager at Editorial Manager® and clicking the ‘Update My Information' link at the top of the page. For questions related to billing, please contact billing support.

If your institution or institutions have a press office, please notify them about your upcoming paper to help maximize its impact. If they’ll be preparing press materials, please inform our press team as soon as possible -- no later than 48 hours after receiving the formal acceptance. Your manuscript will remain under strict press embargo until 2 pm Eastern Time on the date of publication. For more information, please contact onepress@plos.org.

Kind regards,

Nicola Vasta

Academic Editor

PLOS One

Additional Editor Comments (optional):

Reviewers' comments:

# Editor report: Acceptance letter

**DOI:** 10.1371/journal.pone.0352416.r006

**Authors:** Nicola Vasta

PONE-D-26-04456R2

PLOS One

Dear Dr. Jeong,

I'm pleased to inform you that your manuscript has been deemed suitable for publication in PLOS One. Congratulations! Your manuscript is now being handed over to our production team.

At this stage, our production department will prepare your paper for publication. This includes ensuring the following:

* All references, tables, and figures are properly cited

* All relevant supporting information is included in the manuscript submission,

* There are no issues that prevent the paper from being properly typeset

You will receive further instructions from the production team, including instructions on how to review your proof when it is ready. Please keep in mind that we are working through a large volume of accepted articles, so please give us a few days to review your paper and let you know the next and final steps.

Lastly, if your institution or institutions have a press office, please let them know about your upcoming paper now to help maximize its impact. If they'll be preparing press materials, please inform our press team within the next 48 hours. Your manuscript will remain under strict press embargo until 2 pm Eastern Time on the date of publication. For more information, please contact onepress@plos.org.

You will receive an invoice from PLOS for your publication fee after your manuscript has reached the completed accept phase. If you receive an email requesting payment before acceptance or for any other service, this may be a phishing scheme. Learn how to identify phishing emails and protect your accounts at https://explore.plos.org/phishing.

If we can help with anything else, please email us at customercare@plos.org.

Thank you for submitting your work to PLOS ONE and supporting open access.

Kind regards,

PLOS ONE Editorial Office Staff

on behalf of

Dr. Nicola Vasta

Academic Editor

PLOS One
